# Identifying New Loci and Genes Associated with Feed Efficiency in Broilers

**DOI:** 10.3390/ijms26178492

**Published:** 2025-09-01

**Authors:** Na Luo, Peihao Liu, Limin Wei, Jie Wen, Guiping Zhao, Bingxing An

**Affiliations:** 1 Institute of Animal Sciences of Chinese Academy of Agricultural Sciences, Beijing 100193, China; 2 Hainan Provincial Laboratory Animal Research Center, Sanya Research Institute of Hainan Academy of Agricultural Sciences, Sanya 572025, China

**Keywords:** genome-wide association studies, feed conversion ratio, residual feed intake, longitudinal GWAS (LONG-GWAS), chicken 55K SNP array

## Abstract

Feed efficiency is a key economic trait that affects the cost of production in broiler farming. Reducing broiler feed costs contributes to reducing excessive feed consumption and increasing the productivity of broiler breeding. Therefore, identifying genetic regions associated with feed efficiency is crucial for broiler breeding. In this study, we performed genome-wide association (GWAS) analyses of feed conversion ratio (FCR) and residual feed intake (RFI) traits for four growth cycles (72–81, 81–89, 89–113, and 113–120 days of age) using 55K single-nucleotide microarray genotypic data of 4493 Wenchang chickens from two generations. In the single-trait GWAS, a total of 59 SNPs were identified, and 36 genes were annotated within the ±50 kb regions surrounding candidate loci (including *ABCC6*, *CLDN10*, *DGKB*, *EXT2*, *FOXO1*, *IFT140*, *JAG2*, among others. These candidate loci explained 1.4–7.0% of the phenotypic variance explained, and applying a filtering criterion that required a deleteriousness score greater than 8, one locus-*Gallus gallus* chromosome (GGA) 5:3550350 (chCADD score = 12.51524) was located within intron 3 of *ANOX3*. In the FCR and RFI traits in the longitudinal GWAS (LONG-GWAS) model, 80 SNPs and 191 SNPs were identified, respectively, and a total of 43 genes and 121 genes were annotated. A total of 33 candidate loci were screened by combining the locus deleteriousness scores, and 25 candidate genes were annotated within the upper and lower 50 kb ranges. Through KEGG signaling pathway analysis, it was found that the candidate genes were highly enriched mainly in autophagy, mitochondrial phagocytosis, and other pathways. In conclusion, the SNPs and potential genes identified in this study will be helpful for chicken breeding and provide fundamental data for the genetic basis of chicken feed efficiency-related traits.

## 1. Introduction

Growth and feed efficiency characteristics are significant when examining animal growth. Researchers have discovered numerous quantitative trait loci (QTLs) related to feed efficiency [1,2,3,4,5]. One of the biggest production expenses is feeding, and increasing the efficiency of animal feed helps balance ecological and economic impacts by lowering the amount of food inputs used and the effects they have on the environment [6,7,8].

Modern consumers have high standards for both the growth rates of chickens and the quality of their meat. In Hainan, Wenchang chicken, a native breed of Bantam chicken, is the primary source of chicken meat. As people’s awareness of the source of their food has increased, free-range and organic chickens have gained popularity. But selection and breeding of Hainan Wenchang chickens have not yet taken place. Breeds with low feed costs and high animal productivity must be chosen and bred because the cost of feed for animal production has skyrocketed. Reducing feed consumption during broiler breeding and simultaneously increasing yield can be achieved by selecting the original breed based on feed efficiency and by performing genetically efficient selection for animal production through animal breeding.

Residual feed intake (RFI), a linear combination of feed intake, average daily gain (ADG), and metabolic body weight, is a commonly evaluated measure of feed efficiency [9,10]. It frequently includes some body weight metrics [11]. The feed conversion ratio (FCR) and RFI are the primary metrics used to study feed efficiency. RFI is superior to FCR both phenotypically and genetically, and it is more responsive to genetic selection than FCR [12]. These advantages are independent of ADG [13]. The mathematical equivalent of including RFI-type qualities versus individual component attributes in breeding objectives was shown by Van der Werf [14].

Moreover, different growth phases may have distinct loci regulating growth features; however, certain loci may regulate qualities for the duration of an animal’s life [15]. Therefore, conducting independent genome-wide association studies (GWAS) on feed efficiency traits at each stage makes more sense. When determining whether significant single-nucleotide polymorphisms (SNPs) are related to trait development over time, longitudinal GWAS (LONG-GWAS) consider all time points [16]. This approach is effective for locating consistent and time-dependent loci [17]. Building upon single-trait analysis, a LONG-GWAS was conducted to enhance the power of traditional single-trait GWAS.

Nevertheless, longitudinal feed efficiency traits in broilers with yellow features remain underexplored. Thus, knowing the genetic processes behind the individual differences in feed efficiency attributes could offer fresh perspectives on managing chicken growth and productivity. This study used single-trait GWAS and LONG-GWAS models to perform GWAS of feed efficiency traits based on 4493 Wenchang chickens at various growth stages. This allowed for a thorough analysis of candidate genes and QTL regions related to feed efficiency traits. It is understood that this is the first genomic study of feed efficiency traits based on two models. These results will serve as a foundation for further research on additional longitudinal features in domesticated animals and will clarify the molecular basis of developmental traits in Wenchang chickens.

## 2. Results

### 2.1. Descriptive Statistics of Feed Efficiency Traits

**Figure 1:**
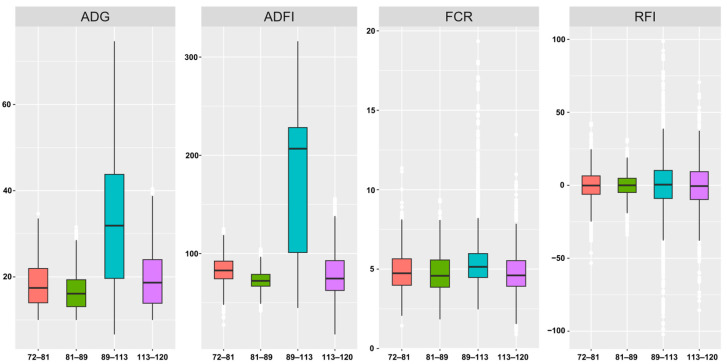
Boxplot of feed efficiency of chickens in different test cycles. From left to right, average daily gain (ADG), average daily feed intake (ADFI), feed conversion ratio (FCR), and residual feed intake (RFI). In the graph, the horizontal coordinates represent the different measurement stages, and the vertical coordinates represent the corresponding measurement values.

We determined descriptive statistics for traits related to feed efficiency traits (Table 1 and Figure 1). The RFI for the different test periods ranged from −102.23 g/d to 98.72 g/d, with an average weight of 1199.68 g at 89 days of age, reaching 1928.96 g at 113 days of age. The mean RFI values being close to zero with ranges spanning negative to positive is expected, as RFI is calculated as residuals from regression, inherently centering around zero. While the chickens digested an average of 73.18–222.25 g of feed per day, the average daily weight gain ranged from 16.54 to 40.95 g. The population coefficients of variation for these traits ranged from 5.64% to 46.82%.

### 2.2. Annotation of Genotype Data After Quality Control

In this study, 55K microarray sequencing was performed on 4493 individual Wenchang chickens. A total of 42,516 high-quality SNP loci were retained after quality control and annotated based on genomic location (Table 2). More than 55.92% of SNPs were in intronic regions, while less than 2% of loci were in the UTR region, and only 4.187% were in the exon region; 9.465% of SNPs were located upstream of the gene, and 9.681% were located downstream of the gene. For SNPs around splice sites (297), only 0.003% and 0.001% were in the splice acceptor region and donor region, respectively; for the variants in the exon region, among them, synonymous mutations accounted for 3.26%, those located in the non-coding exon region accounted for 0.642%, and missense mutations accounted for only 0.308% (268).

### 2.3. SNPs Identified by Single-Trait Genome-Wide Association Analysis

**Figure 2:**
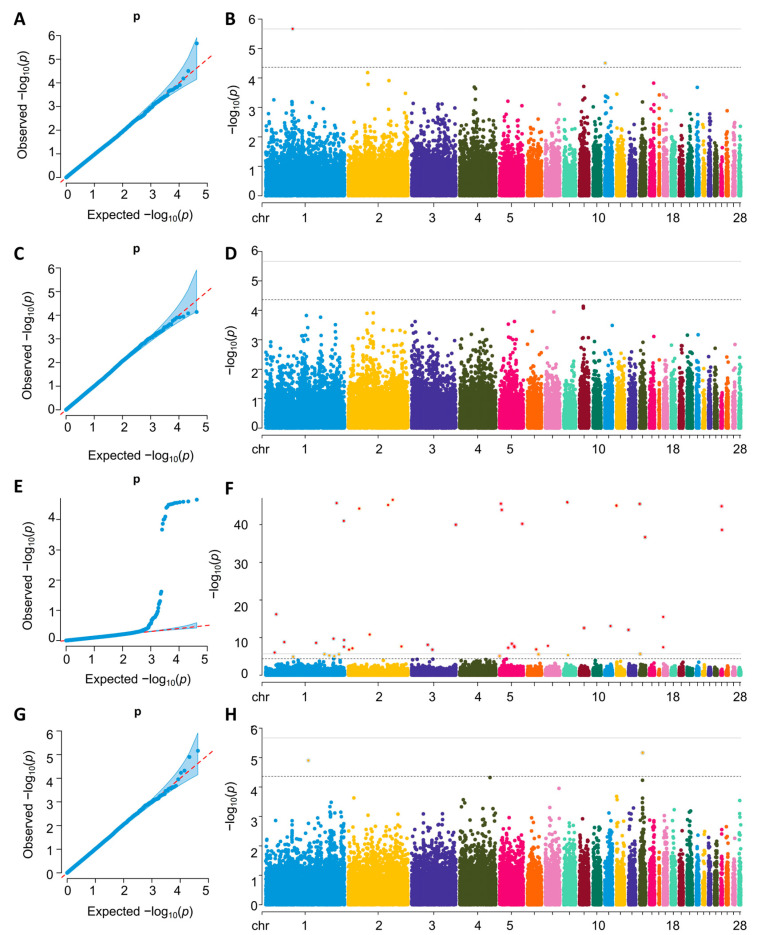
The QQ and Manhattan plot landscapes of the GWAS for 72–81 FCR (**A**,**B**), 81–89 FCR (**C**,**D**), 89–113 FCR (**E**,**F**), and 113–120 FCR (**G**,**H**). The horizontal dashed and solid lines represent the genome-wide significance (*p* = 1.22 × 10^−6^) and suggestive threshold (*p* = 2.44 × 10^−5^), respectively.

**Figure 3:**
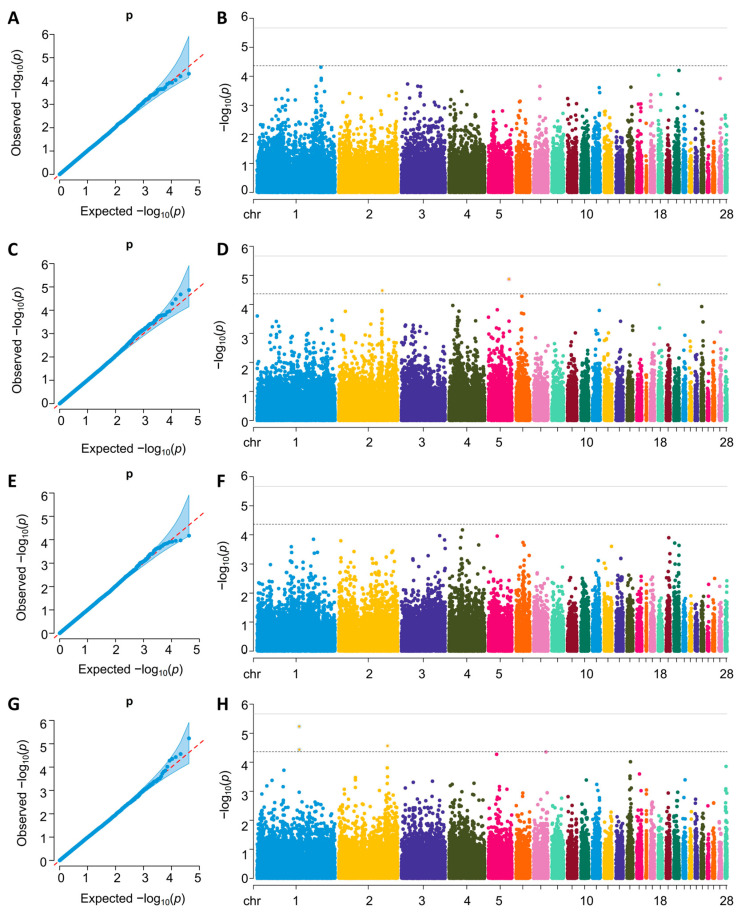
The QQ and Manhattan plot landscapes of the GWAS for 72–81 RFI (**A**,**B**), 81–89 RFI (**C**,**D**), 89–113 RFI (**E**,**F**), and 113–120 RFI (**G**,**H**). The horizontal dashed and solid lines represent the genome-wide significance (*p* = 1.22 × 10^−6^) and suggestive threshold (*p* = 2.44 × 10^−5^), respectively.

The quantile–quantile (Q–Q) and Manhattan plots for the GWAS analysis are shown in Figure 2 and Figure 3. For single-trait GWAS result, the GIFs at each trait ranged from 0.989 to 1.016, which indicated that this association analysis was little affected.

Two significant SNPs (GGA1:67628914 and GGA11:1446218) were identified for FCR during the test period (72 to 81 days of age) and were annotated to two protein-coding genes: *ITPR2* and *CNOT1*. Additionally, two significant SNPs (GGA1:106979821, GGA14:8104013) were identified for the FCR during the test period (113 to 120 days of age) and annotated to the protein-coding genes *ABCC6* and *MRPS6*. Furthermore, fifty significant SNPs were identified for the FCR during the test period (89 to 113 days of age) and were annotated to 29 protein-coding genes, including Diacylglycerol Kinase Beta (*DGKB*) and Forkhead Box O1 (*FOXO1*).

For the RFI trait, three significant SNPs (GGA2:110316845, GGA5:52305921, and GGA18:2827691) were identified during the test period (81 to 89 days of age) and were annotated to the protein-coding genes *RGS20*, *JAG2* and *HS3ST3B1*. Similarly, three significant SNPs (GGA1:107037036, GGA2:123744069, and GGA1:106979821) were identified for the RFI trait during the test period (113 to 120 days of age) and were annotated to the protein-coding genes *MRPS6* and *MMP16*. In total, 37 protein-coding genes were annotated. Notably, there were no significant loci for the single traits 72–81 RFI, 89–113 RFI, and 81–89 FCR. The details of all suggestive SNPs, including their positions in the genome, the nearest reported genes, the MAF, and the *p* values, are listed in Table 3. Recognizing that the majority of our identified SNPs reside in non-coding regions (Table 2), it is crucial to assess their potential functional importance. Intronic and intergenic variants can significantly impact phenotype by altering regulatory elements such as enhancers or splice sites. To prioritize the most likely functional candidates among our non-coding hits, we employed the chCADD score, which predicts the deleteriousness of variants. Using a significance threshold of a chCADD score greater than 8, we filtered our single-trait GWAS results. Notably, the SNP at GGA5:3550350, located within intron 3 of the *ANO3* gene, exhibited a high chCADD score of 12.51524. This high score provides strong evidence that this intronic variant is functionally significant, likely by affecting the regulation of ANO3, and underscores the importance of analyzing non-coding regions in complex trait genetics.

### 2.4. SNPs Identified by Genome-Wide Association Analysis of Longitudinal Traits

**Figure 4:**
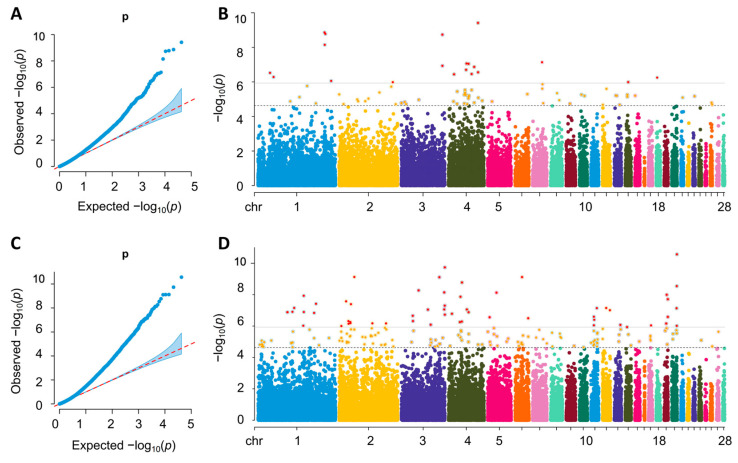
The Manhattan plot landscapes of the LONG-GWAS. The horizontal solid and dotted lines represent the genome-wide significant threshold and genome-wide suggestive significant threshold, respectively. (**A**,**B**) The Q-Q and Manhattan plots of the longitudinal genome-wide association study (GWAS) for FCR (**C**,**D**) The Q-Q and Manhattan plots of the longitudinal genome-wide association study (GWAS) for RFI.

Figure 4 shows the Q-Q and Manhattan plots of the LONG-GWAS analysis for FCR and RFI. The GIFs for the FCR and RFI traits were 1.001 and 1.036, respectively, indicating that the population structure was well controlled. The earlier deviation points in LONG-GWAS QQ plots likely reflect the increased statistical power from modeling time-dependent genetic effects and jointly analyzing correlated phenotypes over time. This approach can detect loci with consistent or cumulative effects over time, leading to earlier departure from the null expectation compared to single-trait GWAS. The Manhattan plots show that there are a total of 80 and 191 genome-wide potential SNPs associated with the FCR and RFI traits. Among these traits, the LONG-GWAS results of FCR and RFI co-identified three loci, GGA1:147675292, GGA1:147675536, and GGA4:75535115. These three loci were annotated to ATP-Binding Cassette Subfamily C Member 4 (*ABCC4*). Details of SNPs associated with FCR and RFI traits are shown in Supplementary Table S3. Utilizing LONG-GWAS analysis, we conducted locus deleteriousness scoring on the FCR and RFI traits across different cycles. Our findings revealed that 6 and 20 loci, respectively, scored above 8 for deleteriousness in the FCR and RFI traits. These high-scoring loci were collectively annotated to 25 candidate genes.

### 2.5. Kyoto Encyclopedia of Genes and Genomes Pathway Analysis of Candidate Genes Associated with Phenotype

**Figure 5:**
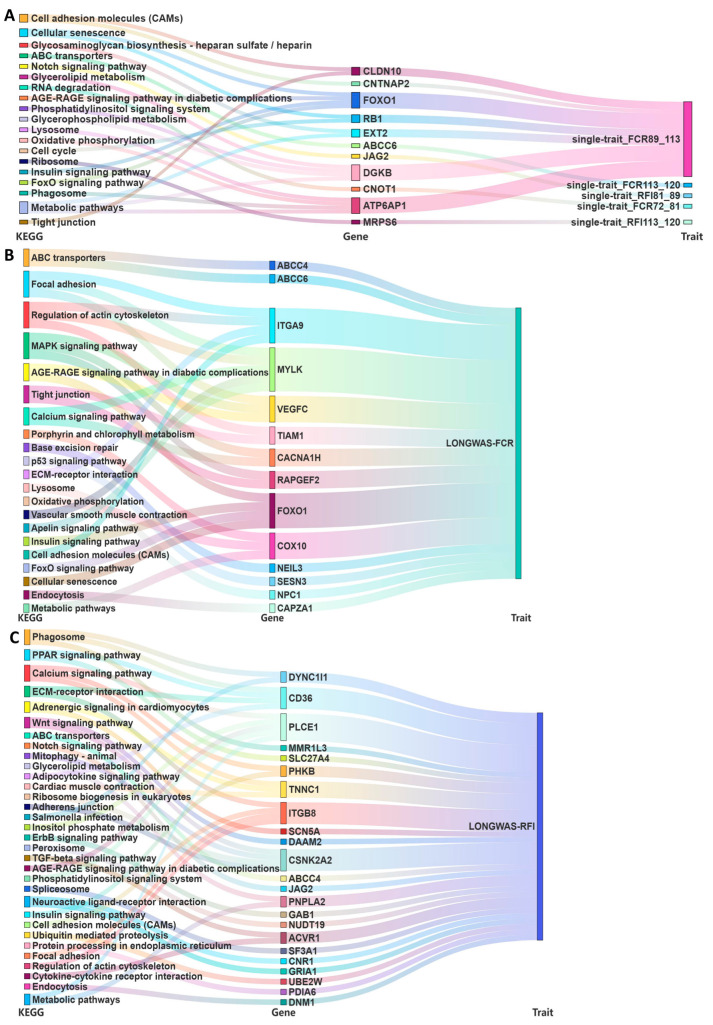
KEGG (Kyoto Encyclopedia of Genes and Genomes) pathway enrichment analyses for GWAS annotation. Sankey diagrams show that potential genes identified via GWAS jointly participated in some pathways. (**A**). KEGG pathway plots for candidate genes identified by single-trait GWAS. (**B**). KEGG pathway plots for candidate genes identified by LONG-GWAS of FCR. (**C**). KEGG pathway plots for candidate genes identified by LONG-GWAS of RFI.

We analyzed a total of 37 protein-coding genes associated with a single trait in the GWAS (Supplementary Table S4, Figure 5A), which were found to be enriched in 36 signaling pathways by KEGG pathway enrichment analysis. Of these pathways, seven were significantly enriched, including the glycosaminoglycan biosynthesis–heparan sulfate/heparin, cellular senescence, phosphatidylinositol signaling system, insulin signaling, mismatch repair, base excision repair, and DNA replication pathways. Notable genes within these pathways included *DGKB,* Heparan Sulfate-Glucosamine 3-Sulfotransferase 3B1 (*HS3ST3B1*)*, FOXO1*, RB Transcriptional Corepressor 1 (*RB1*)*,* Exostosin Glycosyltransferase 2 (*EXT2*), Inositol 1,4,5-Trisphosphate Receptor Type 2 (*ITPR2*), DNA Polymerase Delta 3, Accessory Subunit (*POLD3*), and SHC Adaptor Protein 1 (*SHC1*). For the longitudinal trait analysis, we examined a total of 43 protein-coding genes in the FCR (Supplementary Table S5, Figure 5B), which were enriched in 31 signaling pathways through KEGG pathway enrichment analysis. Eight of these pathways were significantly enriched, including the focal adhesion, regulation of actin cytoskeleton, ABC transporter, AGE-RAGE signaling in diabetic complications, vascular smooth muscle contraction, other glycan degradation, porphyrin and chlorophyll metabolism, and pentose phosphate pathways. Notable genes within these pathways included *ABCC4*, ATP-Binding Cassette Subfamily C Member 6 (*ABCC6*), *FOXO1*, Cytochrome C Oxidase Assembly Factor Heme A: Farnesyltransferase COX10 (*COX10*), Aspartylglucosaminidase (*AGA*), Myosin Light Chain Kinase (*MYLK*), Vascular Endothelial Growth Factor C (*VEGFC*), TIAM Rac1-Associated GEF 1 (*TIAM1*), Integrin Subunit Alpha 9 (*ITGA9*), Phosphofructokinase, Platelet (*PFKP*), and Protein Phosphatase 1 Regulatory Subunit 12A (*PPP1R12A*). For the longitudinal trait RFI analysis (Supplementary Table S6, Figure 5C), we investigated a total of 121 protein-coding genes, which were enriched in 43 signaling pathways by KEGG pathway enrichment analysis. Six of these pathways were significantly enriched, including the phagosome, calcium signaling, basal transcription factor, ABC transporter, Wnt signaling, and glycerolipid metabolism pathways. Notable genes within these pathways included Dynein Cytoplasmic 1 Intermediate Chain 1 (*DYNC1I1*), Macrophage Mannose Receptor 1-like 3 (*MMR1L3*), CD36 Molecule (*CD36*), ATP-Binding Cassette Subfamily B Member 5 (*ABCB5*), *ABCC4*, Troponin C1, Slow Skeletal and Cardiac Type (*TNNC1*), Phosphorylase Kinase Regulatory Subunit Beta (*PHKB*), Casein Kinase 2 Alpha 2 (*CSNK2A2*), Disheveled-Associated Activator Of Morphogenesis 2 (*DAAM2*), General Transcription Factor Iii (*GTF2I*), Inositol-Trisphosphate 3-Kinase A (*ITPKA*), Membrane-Bound O-Acyltransferase Domain-Containing 1 (*MBOAT1*), MMR1L4, Purinergic Receptor P2X 5 (*P2RX5*), Patatin-Like Phospholipase Domain-Containing 2 (*PNPLA2*), Siah E3 Ubiquitin Protein Ligase 1 (*SIAH1*), TATA-Box-Binding Protein-Associated Factor 13 (*TAF13*).

## 3. Discussion

Before delving into the specific genetic loci, it is important to consider the phenotypic trends of feed efficiency observed across the different growth stages. Our descriptive statistics (Figure 1) reveal that the 89–113-day period is the most dynamic phase, characterized by the highest average daily gain (ADG) and feed intake (ADFI). This period represents a critical growth spurt in Wenchang chickens. Correspondingly, the phenotypic variance in RFI was also greatest during this stage, suggesting that genetic differences in metabolic efficiency among individuals are most pronounced when metabolic and growth demands are at their peak.

A key observation from our results is that a large proportion of the trait-associated SNPs were located within intronic or intergenic regions. While variants in exons are often prioritized for their direct impact on protein sequences, the significance of non-coding variants in regulating gene expression is now widely appreciated. Complex quantitative traits, such as feed efficiency, are frequently controlled by subtle variations in the level, timing, or location of gene expression rather than by drastic changes in protein function. Introns, far from being inert spacers, are rich in regulatory elements like enhancers and silencers. An SNP identified by a GWAS in an intron often acts as a marker for a functional regulatory region. It may either be the causal variant itself, altering a transcription factor binding site, or be in strong linkage disequilibrium with the true causal variant nearby. Therefore, the identification of significant intronic SNPs, such as the one in *ANO3* with a high functional prediction score (chCADD > 12), points to these genes being under important regulatory control with respect to feed efficiency. This highlights the necessity of looking beyond the exome to understand the complete genetic architecture of such complex traits.

This observation aligns well with our genetic findings. The single-trait GWAS identified the largest number of significant SNPs for FCR (50 SNPs) precisely within this 89–113 day window. This suggests that the major genetic regulators of feed conversion are most active or detectable during the phase of maximum growth. Therefore, understanding these age-specific phenotypic patterns provides an essential biological context for interpreting our GWAS results and underscores the importance of a longitudinal approach to capture the dynamic nature of feed efficiency.

In poultry breeding, the identification and examination of key SNPs and functional genes that impact the long-term characteristics of broilers are highly profitable. We studied two feed efficiency variables in four growth stages of Hainan Wenchang hens using a single-trait GWAS and a LONG-GWAS. As far as current research indicates, this is the first study to conduct a genome-wide association analysis on feed efficiency attributes measured over several time periods, which provides a more accurate representation of the patterns of growth and development in chickens. These two approaches, however, produced distinct outcomes and had few common loci. The dataset utilized for the single-trait GWAS and LONG-GWAS analyses could be the cause of the disparity. Multiple genes regulate feed efficiency features, a typical phenomenon that cannot be ignored. Each method has a distinct benefit in discovering different loci [18]. For example, whereas a LONG-GWAS enhances the discovery of time-dependent and consistent loci, a single-trait GWAS shows resilience in identifying trait-specific QTLs. It is the main technique for examining longitudinal trait data that is unbalanced, which allows for improved FPR control and breeding value estimation accuracy, both of which increase the effectiveness of GWAS analysis [19,20]. A limited number of genes found in the GWAS are related to feed efficiency, a complex quantitative feature regulated by several genes with subtle effects that prevents a complete understanding of its molecular mechanism. As a result, the examination of the genetic pathways driving feed efficiency features in broilers may be greatly enhanced by combining these two GWAS techniques.

One SNP (GGA1:171954946) located in *FOXO1* was significantly associated with the feed efficiency trait. This finding is strongly supported by the well-established role of *FOXO1* as a vital regulator of muscle mass. Foundational studies in rodent models demonstrated that catabolic stimuli activate the ubiquitin–proteasome system, and that *FOXO1* directly causes muscle atrophy by inducing key atrophy-related genes [21,22]. The timing and mechanism of this activation by the IGF-I-AKT signaling pathway are well characterized in various models, including mouse cell cultures, transgenic mice, and human tissue, establishing *FOXO1′*s role in maintaining muscle homeostasis, controlling differentiation, and specifying muscle fiber type [23,24,25,26,27,28,29,30]. For instance, transgenic mice overexpressing *FOXO1* exhibit significantly reduced muscle mass, confirming its potent catabolic function in vivo [26]. Furthermore, we identified *DGKB*, which was significantly enriched in the glycerophospholipid metabolism pathway. Diacylglycerol kinase converts the bioactive lipid diacylglycerol (DAG) into phosphatidic acid (PA), thus regulating cellular signaling pathways [31]. This pathway is fundamental to fat deposition. The role of diacylglycerol signaling in adipogenesis is well documented, suggesting that variation in *DGKB* could influence the efficiency of energy storage as fat, thus impacting overall feed efficiency.

And one SNP (GGA5:21980526) located in *EXT2* was significantly enriched in the glycosaminoglycan biosynthesis–heparan sulfate/heparin pathway. *EXT2* glycosyltransferase is required for the biosynthesis of heparan sulfate. The *EXT2* complex possesses substantially higher glycosyltransferase activity than *EXT2* alone. Genome-wide resequencing studies in humans have shown that the *EXT2* gene annotated at the rs4755234, rs4755809, and rs6485503 loci affects the growth process of height and body mass [32].

One SNP (GGA14:8104013) was located near *ABCC6* and is enriched in the ABC transporter pathway, where ABC proteins transport a variety of molecules across extracellular and intracellular membranes. Mutations in the *ABCC6* gene can lead to an increased prevalence of coronary artery disease [33]. The *CLDN10* gene at the locus GGA1:147455082 plays a major role in tight-junction-specific occlusion of cell gaps through calcium-independent cell adhesion activity. It is involved in regulating the permeability of paracellular epithelial cells to ions in several organs. The *CLDN10* protein is one of the cytoskeletal proteins and is an important component of intercellular tight junctions [34,35]. Intercellular tight junctions are important channels for maintaining cellular structure as well as intercellular connections and intercellular interactions. The chicken *CNTNAP2* gene is located at GGA2:53866245. It encodes a member of the neurexin superfamily, which functions as a cell adhesion molecule and a receptor in the vertebrate nervous system. This gene has been related to the body mass index of humans [36].

One SNP (GGA1:170070606) was located near *RB1* and has a strong antioxidant effect, and the protein encoded by this gene is a negative regulatory factor for cell cycle. *JAG2* (located GGA5:52305921) is enriched in the Notch signaling pathway, which is an intercellular signal transduction mechanism crucial for normal embryonic development. Members of the Notch gene family encode transmembrane receptors, which are crucial for various cell fate decisions. The protein encoded by this gene is one of several ligands that activate Notch and related receptors. The expression of *JAG2* on the surface of lung adenocarcinoma cells triggers the interaction with Notch receptors and promotes the metastatic potential of these LUAD stem cells [37].

In the LONG-GWAS analysis results, the GGA1:147675292, GGA1:147675536, and GGA4:75535115 genomic loci were associated with FCR and RFI. GGA1:147675292 and GGA1:147675536 were located within the *ABCC4* gene, which is a member of the superfamily of ATP-binding cassette (ABC) transporters. ABC proteins transport various molecules across extra- and intracellular membranes. This protein is a member of the MRP subfamily, which is involved in multidrug resistance, and plays a role in cellular detoxification as a pump for its substrate, organic anions. It may also function in prostaglandin-mediated cAMP signaling in ciliogenesis. The SNP is located at GGA13 at 17.12 Mb, and within the Follistatin-Like 4 (*FSTL4*) gene, which can affect the development of the nervous system in mice [38]. Light stimulation has a great influence on chicken feeding, and the effect of light stimulation acts mainly through neural reflexes; therefore, *FSTL4* may be an important candidate gene for influencing feed efficiency traits in chickens.

Two SNPs associated with feed efficiency are in *EPH Receptor A5* (*EPHA5*), a gene linked to nervous system development and physical activity. Our results in chickens are strongly corroborated by findings in beef cattle, where *EPHA5* has been significantly associated with physical flexibility [39]. This connection is highly relevant to feed efficiency, as physical activity is a major component of maintenance energy requirements and has been estimated to contribute up to 9% of the variation in RFI in cattle [40]. Furthermore, the *Ephrin* receptor family, including *EPHA5*, is widely implicated in shaping neural circuits that control behavior. In livestock, temperament traits, which are governed by the central nervous system, are phenotypically and genetically correlated with feed intake and efficiency [41]. For example, more temperamental, active cattle tend to have poorer feed efficiency. Given that *EPHA5* is highly expressed in the hypothalamus [42]—the brain’s primary center for regulating energy homeostasis and feeding behavior [43,44]—it represents a compelling candidate gene whose variation could influence feed efficiency through centrally mediated effects on both activity and metabolism.

A primary goal of identifying trait-associated SNPs is their application in genetic improvement programs. The significant markers identified in this study, particularly those with high functional scores (e.g., chCADD > 8) within or near key genes like *FOXO1* or *EPHA5*, could be directly utilized in Marker-Assisted Selection (MAS). For instance, breeders could select for favorable alleles at these specific loci to make targeted, rapid improvements in feed efficiency. However, given that feed efficiency is a complex, polygenic trait controlled by numerous genes of small effect, a more powerful application of our findings is through genomic selection (GS). The genome-wide markers from the 55K SNP array provide a foundation for building robust GS prediction models. The key loci discovered in our study can be assigned greater weight within these models to potentially increase the accuracy of Genomic Estimated Breeding Values (GEBVs). Therefore, our results provide foundational data not only for targeted MAS but, more importantly, for implementing advanced genomic selection strategies in the Wenchang chicken breeding program.

## 4. Materials and Methods

### 4.1. Ethics Statement

This study was carried out in accordance with the Guidelines for the Utilization of Experimental Animals set forth by the Ministry of Science and Technology (Beijing, China). All experimental protocols were approved by the Sanya Research Institute (responsible for animal welfare issues), Hainan Academy of Agricultural Sciences (Sanya, China) (approval number: HNSYY20230203).

### 4.2. Animals and Sample Collection

The study utilized chickens sourced from rapidly developing chickens that were chosen by Hainan (Tanniu) Wenchang Chicken Co., Ltd. (Hainan, China) for two generations based on enhanced growth rate characteristics. During the test period (72 to 120 days of age), the measurement of feed efficiency characteristics was conducted on chickens from generations 18 to 19 in two separate batches. The four growth cycles (72–81, 81–89, 89–113, and 113–120 days of age) were selected based on the typical physiological growth curve and production management stages of Wenchang chickens, covering the early, intermediate, and late growth periods, which allowed us to capture stage-specific and lifetime feed efficiency variations. The chickens chosen for individual cage nourishment underwent two generations of selection, where they had unrestricted access to food and water. Details of the feeding protocol, including diet composition, feeding schedule, and management conditions, are provided in Supplementary Table S1. Their weights were measured at the start of the experiment when they were 72, 81, 89, 113, and 120 days old. Blood samples of about 1 mL for DNA analysis were collected at 115 days of age via wing vein punctures using syringes and stored in K_3_ EDTA tubes.

### 4.3. Phenotyping

Feed efficiency traits were calculated at the beginning and end of the per-test period (72–81, 81–89, 89–113, and 113–120), including average daily feed efficiency (ADFI), ADG, FCR, and RFI. The ADFI was calculated using the total feed efficiency of each broiler during each test period. The FCR is defined as the ratio of total feed intake to total body weight gain during each experimental period. RFI was estimated as the difference between the observed and the predicted ADFI. RFI was residual from the following model:

$$ADFI=\mu +batch+sex+\beta_{1}MWT+\beta_{2}ADG+e$$

where *µ* is the intercept, batch and sex are fixed effects, MWT represents the metabolic body weight at mid-test, ADG is as described above, *β*_1_ and *β*_2_ are partial regression coefficients, and *e* is the residual. The phenotypes for all traits were quality-controlled, and the specific number of individuals per trait after quality control is shown in Supplementary Table S2. Prior to the association analyses, all phenotypic data for FCR and RFI were assessed for normality. The Shapiro–Wilk test was performed, and normality was also visually inspected using histograms and quantile–quantile (Q-Q) plots (Supplementary Figures S1 and S2). As a result, when several points are set at a favorable location and the angle line is a reference line, an approximate rectangular distribution appears at the same time. This is our representation of the numerical approximation of the correct distribution.

### 4.4. Genotyping and Quality Control

A TIANamp Blood DNA Kit (Tiangen Biotech Co., Ltd., Beijing, China) was utilized to extract genomic DNA from blood samples, and DNA of excellent quality, with an A260/280 ratio ranging from 1.8 to 2.0, was selected for subsequent analysis. A total of 4493 broilers, consisting of 3640 males and 853 females, were genotyped using the custom 55K SNP array (Beijing Compass Biotechnology Co., Ltd., Beijing, China), which was developed according to the *Gallus_gallus* 6.0 assembly and comprises 52,060 SNPs [45]. The raw sequence data reported in this paper can be publicly accessed at https://bigd.big.ac.cn/gsa/browse/CRA016976 (accessed on 13 August 2025). These broilers belonged to two generations, namely generations 18 and 19. All individuals were utilized for genotype imputation in the designated panel. PLINK (version 1.9) software was utilized for quality control to guarantee the accuracy of the genotypic data [46]. The quality control criteria included a target panel with a minimum individual call rate of 90%, a minimum SNP call rate of 90%, and a minimum minor allele frequency (MAF) of 0.05. Additionally, all SNPs located on the sex chromosomes were disregarded. In the end, there were 42,516 autosome variations and 4493 broilers left for additional examination.

Based on the different locations of the SNPs, the filtered SNPs were annotated using SnpEff v5.0 software [47], whose annotation files were obtained from the ENSEMBL database. Based on their different genomic locations, SNPs were categorized into 10 classes, including 3′ and 5′ UTR regions, upstream and downstream regions, exon and intron regions, splice regions, and intergenic regions. We evaluated the harmfulness of SNP loci through the chCADD score, with a score greater than 8 as the criterion. The chCADD scores can be obtained from OSFHOME (https://osf.io/8gdk9/ (accessed on 13 August 2025)).

### 4.5. Single-Trait Genome-Wide Association Study

The GWAS for FCR and RFI traits was conducted using a univariate linear mixed model (LMM) implemented in GEMMA version 0.98.1 software (https://github.com/genetics-statistics/GEMMA/releases (accessed on 13 August 2025)) [48]. The model for FCR and RFI traits at different stages included the incorporation of batch and sex as fixed effects. The following statistical model was utilized:

y=Xb+Sβ+u+e

In this equation, *y* represents a vector containing phenotypic values, *X* represents the design matrix for fixed effects, b represents the vector of effect estimates including the intercept, *S* represents a vector containing genotypes coded as 0/1/2 for a specific SNP, *β* represents the effect of allele substitution on the fitted SNP, *u* represents a vector of random genetic effects of animals, and *e* represents a vector of residuals. The GenABEL R package (v1.8) [49] was used to calculate the genomic inflation factor (GIF). In this study, a total of 40,942 unaffiliated SNPs were used to establish *p*-value thresholds. To identify significant SNPs while controlling for multiple testing, two significance thresholds were established based on the effective number of independent SNPs (n = 40,942). Following the standard practice in GWAS, an SNP was defined as having genome-wide significance if its *p*-value was below the Bonferroni-corrected threshold of 1.22 × 10^−6^ (0.05/40,942), which strictly controls the family-wise error rate. Additionally, a more lenient suggestive significance threshold was set at 2.44 × 10^−5^ (1/40,942). This threshold was used to identify loci of potential interest that warrant further investigation. The Genetic Type 1 Error Calculator (GEC, https://pmglab.top/gec/#/download, accessed on 13 August 2025) (v1.0) was used to compute the effective number of independent SNPs [50,51]. The CMplot packages [52] in R v4.2.1 were utilized to generate GWAS Manhattan and QQ plots.

### 4.6. LONG Genome-Wide Association Study

A GWAS was conducted on a total of 4493 individuals, encompassing two generations, to analyze phenotypic traits. The analysis of genetic associations for characteristics was conducted using a random regression model implemented in Genomic Multivariate Analysis Tools (LONG-GWAS) software, version 1.01 [19,20,53].

The given statistical model can be represented in the following manner:

$$y_{i}(t)=\mu (t)+x_{i}SNP(t)+a_{i}(t)+p_{i}(t)+e_{i}(t)$$

where *y_i_* is the vector of phenotypic observations for the *i*-th individual. *x_i_* is a genotype indicator that is given values of 0, 1, and 2 for genotypes *aa*, *Aa*, and *AA*, respectively. *a_i_* is the vector of random regression coefficients for the additive genetic effects of the i-th individual. *p_i_* is the vector of random regression coefficients for the permanent environmental effects of the *i*-th individual. *e_i_* is the vector of random residual errors. *SNP*(*t*) denotes the time-varying additive effect for each marker and can be represented as a linear regression using a set of previously mentioned basic functions.

$$SNP(t)=\sum_{k=0}^{nf}\eta_{k}\Phi_{k}(t),$$

The value of the *k*th basis function at time *t* is represented by *Φ_k_(t),* while *η_k_* denotes the fixed regression coefficient for additive SNP, and *nf* indicates the order of basic functions for the time-varied SNP effect. The model is established assuming that the order of the time-varying population mean and SNP effects is the same.

In the same way, the fGWAS-F model is expressed in the following manner.

$$y_{il}(t)=\mu (t)+SNP_{l}(t)+a_{i}(t)+pe_{i}(t)+e_{il}(t),$$

where

$$SNP_{l}(t)=\sum_{k=0}^{nf}\lambda_{lk}\Phi_{k}(t).$$

The time-varying effect for genotype *l* (*AA*, *Aa*, and *aa*) of each marker is represented by *SNPl* (*t*), and *λlk* is the *k*th fixed regression coefficient for genotype *l*. The fGWAS-F model allows for the deduction of the time-varying additive genetic effect, dominance genetic effect, and additive genetic variance of each SNP according to [54]:

$$add(t)=\frac{{SNP}_{AA}(t)-{SNP}_{aa}(t)}{2},\;dom(t)={SNP}_{Aa}(t)\;-\;add(t)$$

$$and\;\sigma_{a,SNP}^{2}(t)=2pq(add(t)+dom(t)(q-p){)}^{2},$$

where *p* and *q* represent the allele frequencies for every locus.

### 4.7. Functional Annotation

After applying the *p* value threshold, we screened the noteworthy SNPs associated with characteristics and subsequently employed Ensembl–BioMart to align these SNPs with the chicken reference genome available at http://useast.ensembl.org/Gallus_gallus/Info/Index (accessed on 13 August 2025). The Variant Effect Predictor (VEP) was used to annotate candidate genes. For each significant SNP, genes located within a 100 kb window (±50 kb from the SNP) were considered to be potential candidate genes. This range was chosen based on the typical linkage disequilibrium (LD) decay distance in the chicken genome and is a common approach to capturing genes that may be affected by the causal variant in LD with the tag SNP. The annotation was based on the GRCg6a (GCA_000002315.5) assembly endorsed by Ensembl (http://useast.ensembl.org/Gallus_gallus/Info/Index (accessed on 13 August 2025)). Kyoto Encyclopedia of Genes and Genomes (KEGG) pathway enrichment analysis was performed using KOBAS version 3.0 (http://bioinfo.org/kobas (accessed on 13 August 2025)). The level of significance was fixed at *p*  <  0.05.

## 5. Conclusions

In conclusion, by integrating single-trait and longitudinal GWAS, this study successfully identified novel genetic loci associated with feed efficiency in Wenchang chickens. Our analysis highlighted high-confidence candidate genes with key biological functions, including *FOXO1*, *DGKB*, *ABCC4*, and *EPHA5*, which are linked to muscle metabolism, energy expenditure, and transport processes. These identified SNPs and genes serve as valuable molecular markers to accelerate genetic improvement of feed efficiency through genomic selection. Future priorities include the functional validation of these candidates to confirm their physiological roles. Ultimately, translating these genomic discoveries into tangible genetic gains will pave the way for a more sustainable and economically viable broiler industry, demonstrating how modern genomics can address critical challenges in global food production.

## Figures and Tables

**Table 1:** Descriptive statistics for feed efficiency traits in different stages.

Traits (g/d)	N	Mean	SD	Max	Min	CV (%)
72–81 ADG	788	18.32	5.28	34.67	10	29
72–81 ADFI	937	83.02	14.35	125	27.44	17
72–81 FCR	787	4.92	1.31	11.36	1.44	27
72–81 RFI	787	−6.35 × 10^−11^	11.69	42.53	−53.32	/
81–89 ADG	1540	16.54	4.20	31.56	10	25
81–89 ADFI	1877	73.18	10.23	104.67	41.67	14
81–89 FCR	1530	4.74	1.20	9.36	1.83	25
81–89 RFI	1530	−1.05 × 10^−11^	8.27	31.33	−33.97	/
89–113 ADG	3698	32.57	13.78	74.67	6.65	42
89–113 ADFI	3592	173.98	66.11	316.11	44.65	38
89–113 FCR	3592	5.48	1.70	19.35	2.47	31
89–113 RFI	3592	1.84 × 10^−11^	19.17	98.72	−102.23	/
113–120 ADG	1482	19.47	6.61	40.44	10	34
113–120 ADFI	2071	79.93	25.53	155.78	17.67	32
113–120 FCR	1481	4.75	1.31	13.47	0.89	28
113–120 RFI	1481	2.30 × 10^−11^	17.33	70.56	−85.70	/

Abbreviations: 72–81 ADG, average daily gain at 72–81 days of age; 72–81 ADFI, average daily feed efficiency at 72–81 days of age; 72–81 FCR, feed conversion ratio at 72–81 days of age; 72–81 RFI, residual feed intake at 72–81 days of age; 81–89 ADG, average daily gain at 81–89 days of age; 81–89 ADFI, average daily feed efficiency at 81–89 days of age; 81–89 FCR, feed conversion ratio at 81–89 days of age; 81–89 RFI, residual feed intake at 81–89 days of age; 89–113 ADG, average daily gain at 89–113 days of age; 89–113 ADFI, average daily feed efficiency at 89–113 days of age; 89–113 FCR, feed conversion ratio at 89–113 days of age; 89–113 RFI, residual feed intake at 89–113 days of age; 113–120 ADG, average daily gain at 113–120 days of age; 113–120 ADFI, average daily feed efficiency at 113–120 days of age; 113–120 FCR, feed conversion ratio at 113–120 days of age; 113–120 RFI, residual feed intake at 113–120 days of age; N, number of samples; SD, standard deviation; CV, coefficient of variance.

**Table 2:** Annotation of genotype data after quality control.

Type (Alphabetical Order)	Count	Percentage
3_prime_UTR_variant	782	0.009
5_prime_UTR_premature_start_codon_gain_variant	25	0.00029
5_prime_UTR_variant	125	0.00144
downstream_gene_variant	8382	0.09648
intergenic_region	16,828	0.1937
intron_variant	48,581	0.55919
missense_variant	268	0.00308
non_coding_transcript_exon_variant	558	0.00642
splice_acceptor_variant	3	0.00003
splice_donor_variant	1	0.00001
splice_region_variant	296	0.00341
stop_gained	2	0.00002
synonymous_variant	2832	0.0326
upstream_gene_variant	8195	0.09433

**Table 3:** Information on SNPs associated with feed efficiency traits based on single-trait model.

Traits	GGA	Position	Allele	AF	Beta	*p*	PVE	Gene	Ensembl ID	CADD
72–81 FCR	11	1446218	T/A	0.039	−0.7419951	3.14011 × 10^−5^	0.025887031	*CNOT1*	ENSGALG00000002301	4.12349
72–81 FCR	1	67628914	T/C	0.35	−0.3458966	2.14648 × 10^−6^	0.035913685	*ITPR2*	ENSGALG00000014071	0.00395
89–113 FCR	6	22856589	T/C	0.071	−0.3941262	1.37804 × 10^−7^	0.008497121	*ADGRA1*	ENSGALG00000007045	0.41839
89–113 FCR	7	6630777	G/T	0.165	0.3401085	1.60176 × 10^−8^	0.009312092	*AHR1B*	ENSGALG00000004322	0.16682
89–113 FCR	5	3550350	G/T	0.384	−0.4573349	2.92904 × 10^−46^	0.072729824	*ANO3*	ENSGALG00000013311	12.51524
89–113 FCR	1	22514424	A/C	0.069	0.4141535	9.66446 × 10^−7^	0.007064396	*ATP6AP1*	ENSGALG00000008836	3.72873
89–113 FCR	1	147455082	G/A	0.484	0.1868363	2.58236 × 10^−6^	0.006392649	*CLDN10*	ENSGALG00000019114	0.81705
89–113 FCR	2	27469326	A/C	0.39	−0.4518481	5.48257 × 10^−45^	0.07039052	*DGKB*	ENSGALG00000033561	2.15319
89–113 FCR	5	21980526	G/A	0.357	0.2408767	5.26196 × 10^−8^	0.008563368	*EXT2*	ENSGALG00000031542	0.77067
89–113 FCR	1	171954946	T/G	0.159	0.2839152	9.51733 × 10^−6^	0.00574995	*FOXO1*	ENSGALG00000017034	2.88924
89–113 FCR	1	26392940	T/C	0.461	−0.3662863	6.27082 × 10^−17^	0.022365852	*FOXP2*	ENSGALG00000009424	1.46472
89–113 FCR	1	177838774	G/A	0.388	−0.4592473	1.88762 × 10^−46^	0.073054161	*GPR12*	ENSGALG00000017102	6.93379
89–113 FCR	14	14152014	A/G	0.419	−0.4196301	2.26874 × 10^−37^	0.056652498	*IFT140*	ENSGALG00000009318	5.986
89–113 FCR	5	57035557	T/C	0.436	−0.4399652	6.21205 × 10^−41^	0.062907477	*MDGA2*	ENSGALG00000012228	1.62758
89–113 FCR	1	183820939	A/C	0.436	0.1851372	2.7826 × 10^−6^	0.006350488	*MMP27*	ENSGALG00000019060	1.43259
89–113 FCR	6	28586738	A/G	0.169	0.2801326	2.83443 × 10^−6^	0.006415069	*NHLRC2*	ENSGALG00000008946	3.70314
89–113 FCR	5	36810052	C/T	0.3	0.2674278	1.98998 × 10^−8^	0.009124893	*NKX2−1*	ENSGALG00000037632	1.05414
89–113 FCR	1	196418182	G/A	0.259	0.3011632	4.69091 × 10^−10^	0.011214515	*P2RY6*	ENSGALG00000017327	1.49934
89–113 FCR	5	298547	A/G	0.073	−0.3716725	8.55429 × 10^−6^	0.006026163	*PGA5*	ENSGALG00000039242	2.29974
89–113 FCR	1	195873238	A/C	0.408	−0.4398747	1.07204 × 10^−41^	0.064351973	*POLD3*	ENSGALG00000017307	2.20877
89–113 FCR	1	196207597	G/C	0.288	0.2623651	2.88412 × 10^−8^	0.008916544	*RAB6A*	ENSGALG00000017320	4.86582
89–113 FCR	1	170070606	G/C	0.162	0.3721619	2.05114 × 10^−10^	0.011752907	*RB1*	ENSGALG00000016997	2.29389
89–113 FCR	25	2987929	A/G	0.519	0.4489232	2.64559 × 10^−39^	0.047156239	*SHC1*	ENSGALG00000039775	2.42158
89–113 FCR	14	750730	T/C	0.388	−0.4576204	3.2049 × 10^−46^	0.072646283	*SHISA9*	ENSGALG00000035799	2.72137
89–113 FCR	5	30932965	C/T	0.271	0.2923482	4.41261 × 10^−9^	0.009977342	*SPRED1*	ENSGALG00000028203	0.64103
89–113 FCR	1	46624790	A/G	0.353	0.2596588	1.599 × 10^−9^	0.010485056	*TMPO*	ENSGALG00000011504	6.83232
89–113 FCR	3	52101965	G/A	0.066	0.4624581	1.73306 × 10^−7^	0.00804205	*TULP4*	ENSGALG00000037377	1.36685
89–113 FCR	2	1951183	G/C	0.161	0.3088039	1.62009 × 10^−7^	0.008010282	*VIPR1*	ENSGALG00000005259	7.10688
89–113 FCR	2	10466524	A/C	0.111	0.3675874	7.28495 × 10^−8^	0.008488573	*WDR37*	ENSGALG00000006749	0.2238
89–113 FCR	5	5671784	A/G	0.396	−0.4519878	1.24313 × 10^−44^	0.069699203	*WT1*	ENSGALG00000012115	6.0281
113–120 FCR	14	8104013	G/A	0.411	0.2407186	6.91816 × 10^−6^	0.014425553	*ABCC6*	ENSGALG00000038152	1.79817
113–120 FCR	1	106979821	C/T	0.297	−0.2535606	1.25899 × 10^−5^	0.015346278	*MRPS6*	ENSGALG00000027579	0.25737
81–89 RFI	18	2827691	T/C	0.101	−2.33695	2.09453 × 10^−5^	0.013461153	*HS3ST3B1*	ENSGALG00000001371	2.81062
81–89 RFI	5	52305921	A/T	0.397	−1.471009	1.36042 × 10^−5^	0.014130899	*JAG2*	ENSGALG00000011696	3.88963
81–89 RFI	2	110316845	T/C	0.307	−1.470263	3.34978 × 10^−5^	0.01283052	*RGS20*	ENSGALG00000025941	0.47986
113–120 RFI	2	123744069	G/A	0.375	−2.928533	2.74233 × 10^−5^	0.013398714	*MMP16*	ENSGALG00000032031	1.16648
113–120 RFI	1	106979821	C/T	0.297	−0.2535606	5.87375 × 10^−6^	0.015654765	*MRPS6*	ENSGALG00000027579	0.25737
113–120 RFI	1	107037036	G/A	0.251	−3.274938	3.72438 × 10^−5^	0.01294475	*MRPS6*	ENSGALG00000027579	1.99

## Data Availability

The data reported in this paper have been deposited in OMIX, China National Center for Bioinformation/Beijing Institute of Genomics, Chinese Academy of Sciences (https://ngdc.cncb.ac.cn/omix accession no.OMIX007730 (accessed on 13 August 2025)) [55].

## Supplementary Materials

The following supporting information can be downloaded at: https://www.mdpi.com/article/10.3390/ijms26178492/s1.

## Abbreviations

The following abbreviations are used in this manuscript:

ABC	ATP-binding cassette
ABCC4	ATP-Binding Cassette Subfamily C Member 4
ABCB5	ATP-Binding Cassette Subfamily B Member 5
ABCC6	ATP-Binding Cassette Subfamily C Member 6
ADFI	average daily feed efficiency
ADG	average daily gain
AGA	Aspartylglucosaminidase
chCADD	chicken Combined Annotation-Dependent Depletion
COX10	Cytochrome C Oxidase Assembly Factor Heme A: Farnesyltransferase COX10
CSNK2A2	Casein Kinase 2 Alpha 2
DAAM2	Disheveled-Associated Activator of Morphogenesis 2
DGKB	Diacylglycerol Kinase Beta
DYNC1I1	Dynein Cytoplasmic 1 Intermediate Chain 1
EPHA5	EPH Receptor A5
EXT2	Exostosin Glycosyltransferase 2
FCR	feed conversion ratio
FOXO1	Forkhead Box O1
FSTL4	Follistatin Like 4
GEC	Genetic Type 1 Error Calculator
GGA	*Gallus gallus* chromosome
GIF	genomic inflation factor
GTF2I	General Transcription Factor Iii
GWAS	genome-wide association
HS3ST3B1	Heparan Sulfate-Glucosamine 3-Sulfotransferase 3B1
ITGA9	Integrin Subunit Alpha 9
ITPKA	Inositol-Trisphosphate 3-Kinase A
ITPR2	Inositol 1,4,5-Trisphosphate Receptor Type 2
KEGG	Kyoto Encyclopedia of Genes and Genomes
LMM	linear mixed model
LONG-GWAS	longitudinal GWAS
MAF	minimum minor allele frequency
MBOAT1	Membrane-Bound O-Acyltransferase Domain-Containing 1
MMR1L3	Macrophage Mannose Receptor 1-like 3
MYLK	Myosin Light Chain Kinase
P2RX5	Purinergic Receptor P2X 5
PFKP	Phosphofructokinase, Platelet
PHKB	Phosphorylase Kinase Regulatory Subunit Beta
PNPLA2	Patatin-Like Phospholipase Domain-Containing 2
POLD3	DNA Polymerase Delta 3, Accessory Subunit
PPP1R12A	Protein Phosphatase 1 Regulatory Subunit 12A
QTL	quantitative trait locus
Q–Q	quantile–quantile
RB1	RB Transcriptional Corepressor 1
RFI	residual feed intake
SHC1	SHC Adaptor Protein 1
SIAH1	Siah E3 Ubiquitin Protein Ligase 1
SNPs	Single-nucleotide polymorphisms
TAF13.	TATA-Box-Binding Protein-Associated Factor 13
TIAM1	TIAM Rac1-Associated GEF 1
TNNC1	Troponin C1, Slow Skeletal and Cardiac Type
VEGFC	Vascular Endothelial Growth Factor C
