# Supplementary figures and images for: Identifying New Loci and Genes Associated with Feed Efficiency in Broilers
Source: Int J Mol Sci. 2025 Sep 1;26(17):8492. doi: 10.3390/ijms26178492 (PMC12429723; doi:10.3390/ijms26178492)

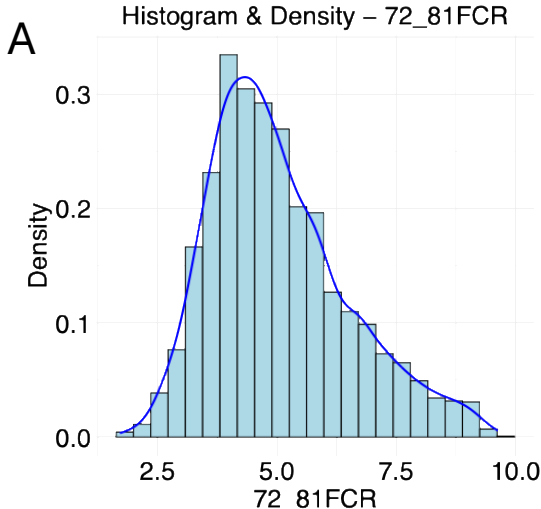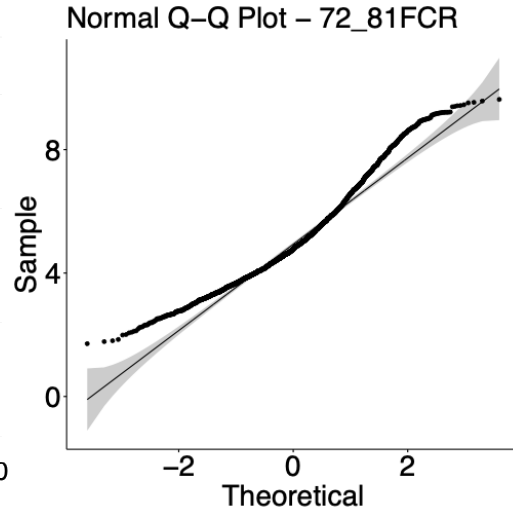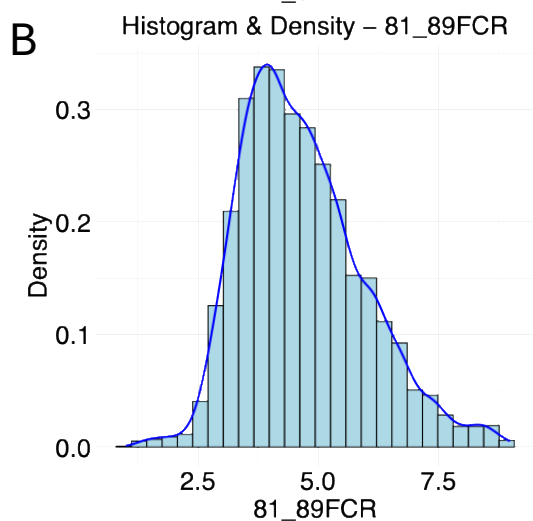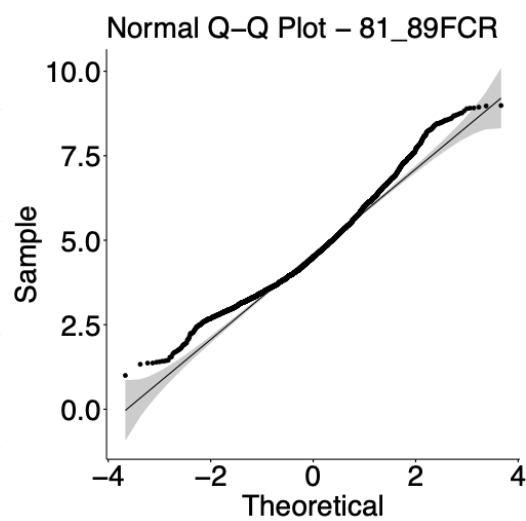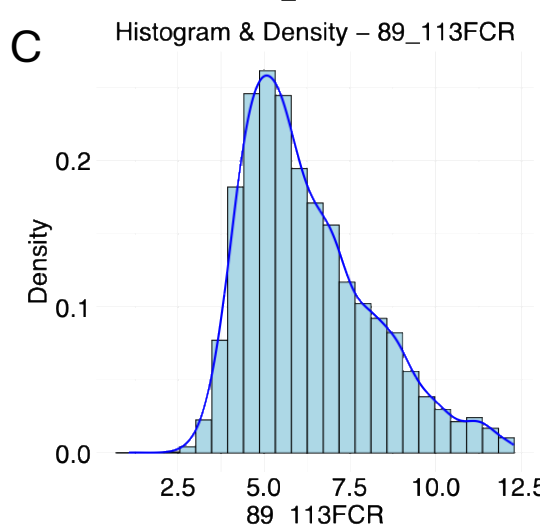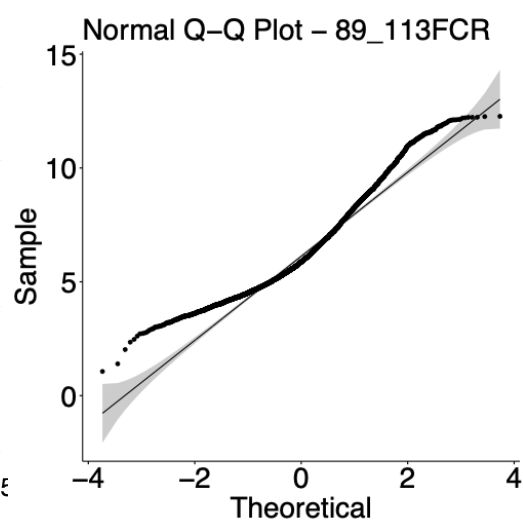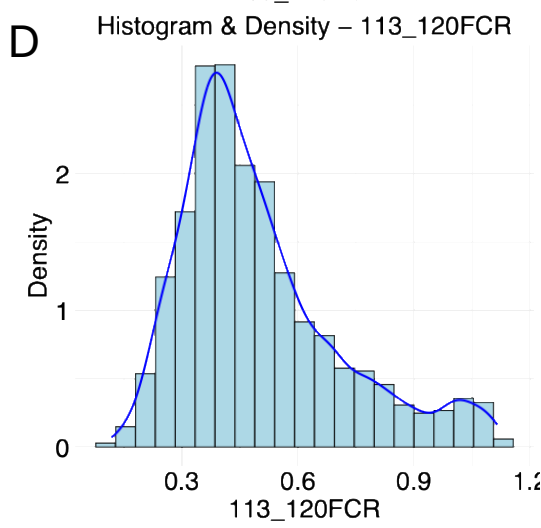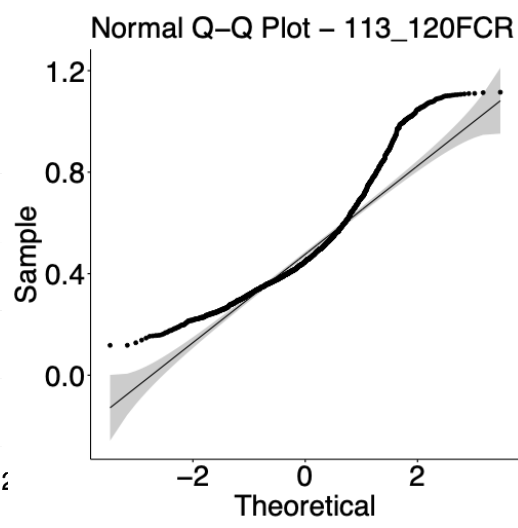

Supplement: Supplementary file 1 [file ijms-26-08492-s001.zip › Additional Figure S1.pdf]

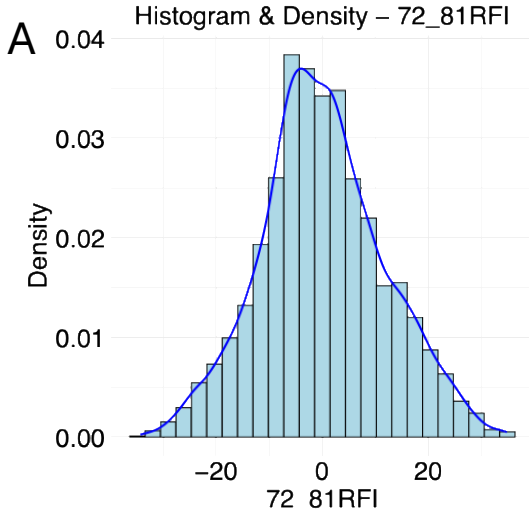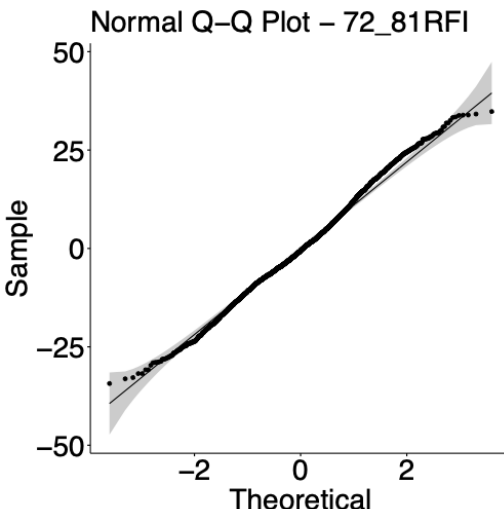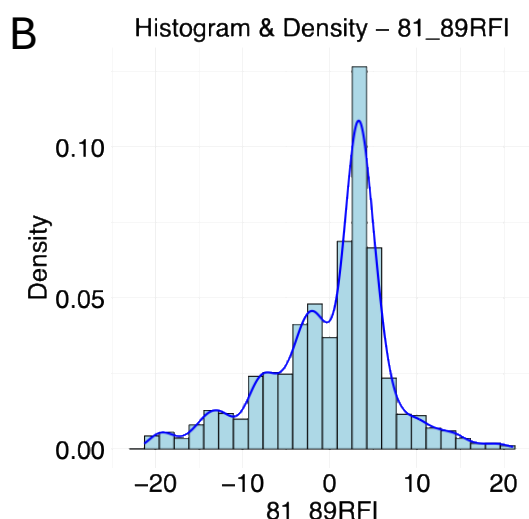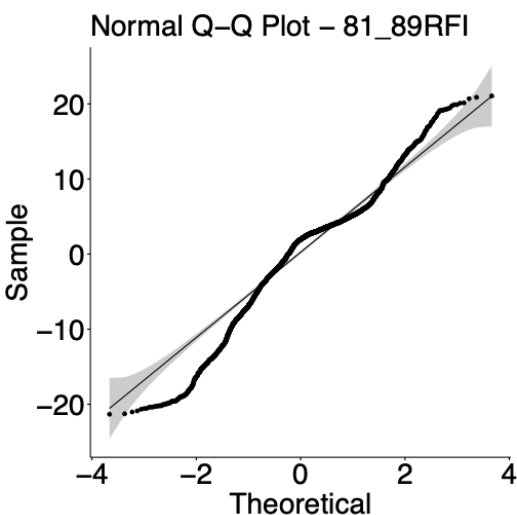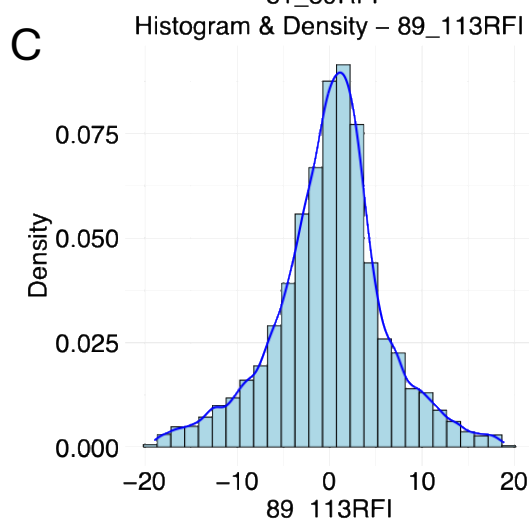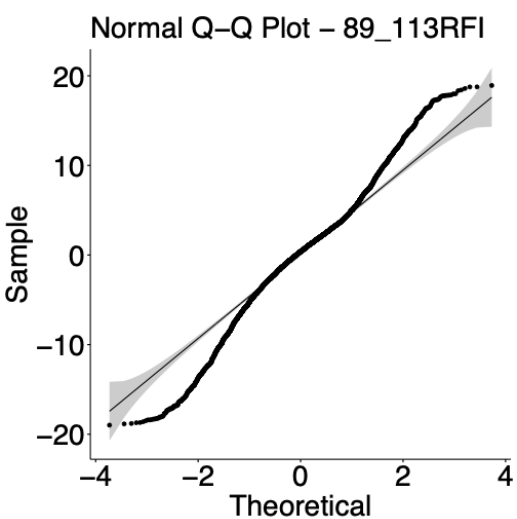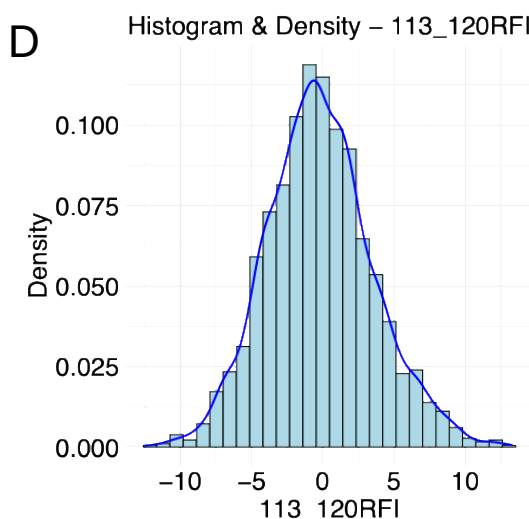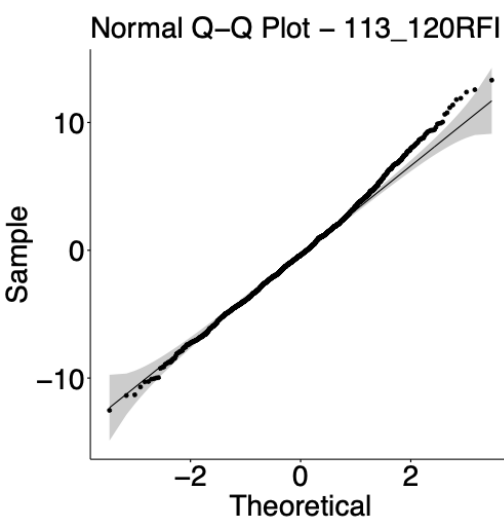

Supplement: Supplementary file 1 [file ijms-26-08492-s001.zip › Additional Figure S2.pdf]
